# Critical Analysis of Forest Degradation in the Southern Eastern Ghats of India: Comparison of Satellite Imagery and Soil Quality Index

**DOI:** 10.1371/journal.pone.0147541

**Published:** 2016-01-26

**Authors:** Andimuthu Ramachandran, Parthasarathy Radhapriya, Shanmuganathan Jayakumar, Praveen Dhanya, Rajadurai Geetha

**Affiliations:** 1 Centre for Climate Change and Adaptation Research, Anna University, Chennai, Tamil Nadu, India; 2 Environmental Informatics and Spatial Modelling Lab (EISML), Department of Ecology & Environmental Sciences, School of Life Sciences, Pondicherry University, Puducherry, India; University of Guelph, CANADA

## Abstract

India has one of the largest assemblages of tropical biodiversity, with its unique floristic composition of endemic species. However, current forest cover assessment is performed via satellite-based forest surveys, which have many limitations. The present study, which was performed in the Eastern Ghats, analysed the satellite-based inventory provided by forest surveys and inferred from the results that this process no longer provides adequate information for quantifying forest degradation in an empirical manner. The study analysed 21 soil properties and generated a forest soil quality index of the Eastern Ghats, using principal component analysis. Using matrix modules and geospatial technology, we compared the forest degradation status calculated from satellite-based forest surveys with the degradation status calculated from the forest soil quality index. The Forest Survey of India classified about 1.8% of the Eastern Ghats’ total area as degraded forests and the remainder (98.2%) as open, dense, and very dense forests, whereas the soil quality index results found that about 42.4% of the total area is degraded, with the remainder (57.6%) being non-degraded. Our ground truth verification analyses indicate that the forest soil quality index along with the forest cover density data from the Forest Survey of India are ideal tools for evaluating forest degradation.

## Introduction

India, a country with rich biodiversity, has different forest types in different climatic zones, each with a unique floristic composition [1, 2]. The forests are mainly distributed in the Himalayas, Western Ghats, Eastern Ghats (EG), and Vindhya ranges. Indian forests have historically experienced a wide variety of management practices because of the differing policies of diverse peoples and governances [2, 3]. In particular, the protected forest reserves of the southern EG, an area in Tamil Nadu [4, 5], had experienced considerable need-based forestry practices (e.g. felling of timber for construction, fuel, or the creation of grazing lands and real estate) for more than 150 years until 1980 [6–10]. As a result, the floristic composition of the forests in the EG underwent massive changes [10, 11], including the emergence of secondary and tertiary vegetation. Furthermore, severe, negative effects on soil health were also reported [8]. Therefore, effective monitoring and inventory strategies are crucial for the continued conservation of these forests.

Currently, the Forest Survey of India (FSI) periodically monitors forest status based on canopy density, which is obtained via satellite data with ground truth verification. Canopy density is classified into four categories: (i) very dense forest (VDF) with >70% canopy density, (ii) moderately dense forest with 40%–70%, canopy density, (iii) open forest with 10%–40% canopy density, and (iv) degraded forest with <10% canopy density [12]. This classification is based on a normalised difference vegetation index (NDVI), which does not distinguish between crown density and ground density [13]. However, research has shown that the NDVI is not detailed enough to provide reliable data and may underestimate the degradation status [14–17]**.** Menon and Bawa [18], who estimated the deforestation rates in India and identified large disparities in the forest definitions, also highlighted the need for improved remote sensing and ground truth verification data to discriminate native and plantation forests. Moreover Aziz et al. [19] clearly inferred that the NDVI could not capture the difference between natural forests and rubber plantations in Malaysian forests.

Soil is a vital component of forest ecosystems and is responsible for processes that support biomass production and carbon sequestration [20]. Forest soil health is sensitive to numerous natural and anthropogenic factors, and a deterioration of soil quality typically leads to a deterioration of site quality [21]. Thus, it is important to establish appropriate soil quality indices for measuring the various properties of soil. A soil quality index is defined as the minimum set of parameters that, when interrelated, provides numerical data on the capacity of a soil to carry out one or more functions. A soil quality indicator is a measurable property that influences the capacity of a soil to carry out a given function [22]. A soil quality index, to some extent, should be use-dependent, so that it can be applicable on a larger scale. SQI will help us to select a minimum set of indicators that can address the overall soil quality of a particular region [23]. Foresters have always relied on their knowledge of the chemical, physical, and biological properties of soils to assess the capacity of a site to support productive forests [24]. Recently, the need to assess soil properties has expanded because of growing public interest in determining the consequences of management practices on the quality of soil relative to the sustainability of forest ecosystem functions in addition to plant productivity [25]. The concept of soil quality includes the assessment of soil properties and processes as they relate to the soil’s ability to function effectively as a component of a healthy ecosystem [26]. The specific functions and subsequent values provided by forest ecosystems are variable and rely on numerous soil properties and processes [27]. Thus, an understanding of soil properties and soil biota is imperative to the accurate classification of the forest degradation level [8].

Forest soil quality assessment is well established in several developed countries, such as the United States [28], New Zealand [29], and numerous European nations [30], including the United Kingdom [31]. In India, because the majority of soil quality studies are performed in agricultural and horticultural sectors [32, 33], very little data are available on forest soil quality and biota [8]. Therefore, the aim of this study was to identify the degradation status of forests in the EG by performing a detailed soil quality assessment, and then using geospatial technology to compare the soil quality results with those obtained from FSI forest density classes.

## Materials and Methods

### Study area

**Fig 1:**
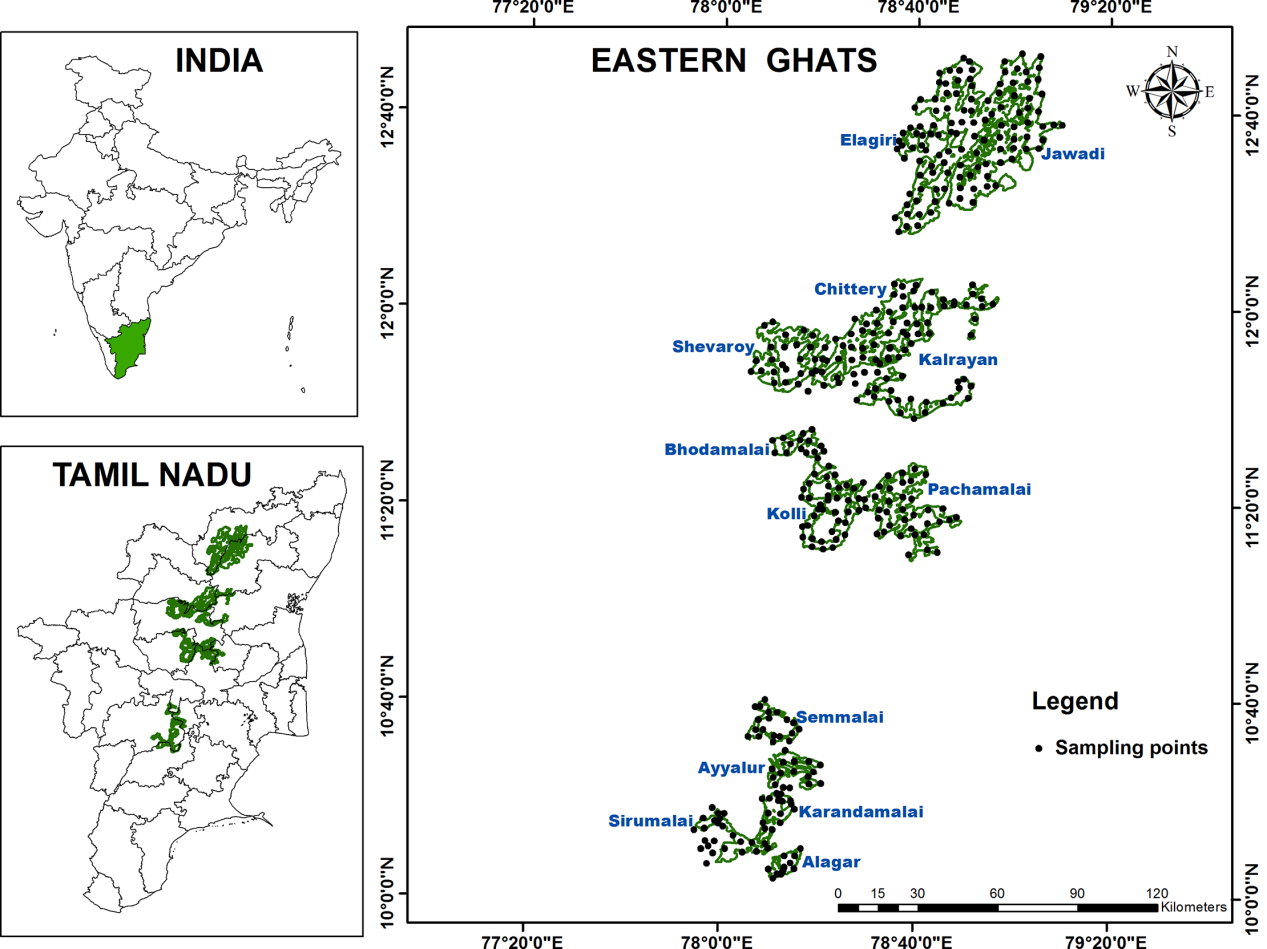
Study area—Eastern Ghats of Southern India.

The EG (between 10°00′00″–13°00′00″N and 77°50′00″–79°10′00″E) is a discontinuous mountain range running almost parallel to the east coast of India and constitutes the watersheds of many rivers. In Tamil Nadu, the EG start from the Jawadi Hills and extend up to the Alagar Hills (Fig 1), comprising 13 major hills in total (Jawadi, Elagiri, Shevaroy, Chitteri, Kalrayan, Bodamalai, Kolli, Pachaimalai, Semmalai, Aiyalur, Karandamalai, Sirumalai, and Alagar). The hills of the EG in Tamil Nadu range from 70 km^2^ to 1860 km^2^ in area, and the altitude in this region ranges from 180 m above mean sea level (MSL) to 1650 m above MSL. The mean minimum and maximum temperatures are 17°C and 33°C, respectively, and the average annual rainfall is 800–1600 mm. The major soil types are entisols, inceptisols, and alfisols. Geologically, the EG consist of charnockite with minor bands of pyroxin granulate and magnetite quartzite. The tributaries of many perennial rivers like the Pennaiyar, Palar, Vellar, Cauvery, and Vaigai originate from these hills. These hills are still rich in biodiversity, despite heavy exploitation, and they contain more than 960 species of angiosperm and gymnosperm. There are many endemic, endangered, and medicinally important species distributed within these hills [34, 35].

The forests are mostly degraded due to the repeated felling of trees in the past; presently, three major types of forests exist, with different compositions and canopy densities, as explained in detail below.

#### Tropical dry evergreen forest

This forest type is referred to locally as ‘shola forest’ and it occurs in six hills: Jawadi, Elagiri, Sherveroy, Kolli, Pachaimalai, and Sirumalai, which are generally located 1000 m above MSL. The general composition includes *Memecylon edule*, *Neolitsea scrobiculata*, *Persea macrantha*, *Memecylon umbellatum*, *Elaeocarpus serratus*, *Syzygium cumini*, *Canarium strictum*, *Artocarpus heterophyllus*, *Artocarpus hirsuta*, and *Alangium salvifolium*, while the dominant shrub species are *Psychotria subintegra*, *Mesa indica*, *Glycosmis mauritiana*, *Phyllanthus wightianus*, *Tarenna asiatica*, *Lantana camara*, and *Clausena dentata*, among others.

#### Tropical dry deciduous forest

This forest type is widely distributed throughout the 13 hills and is found in all types of topography, such as valleys, plateaus, and foothills from 400 m up to 1000 m above MSL. The species composition in the upper and middle slopes consists of *Albizia odoratissima*, *Bridelia retusa*, *Chloroxylon swietenia*, *Terminalia chebula*, *T*. *bellirica*, *Tectona grandis*, *Schleichera oleosa*, *Sapindus emarginatus*, *Emblica officinalis*, *Pterocarpus marsupium*, *Dalbergia latifolia*, *Gyrocarpus asiaticus*, *and Moringa oleifera*, among others, while the dominant shrubs are *G*. *mauritiana*, *L*. *camara*, *Pterolobium hexapetalum*, *Ziziphus oenoplia*, and *Acacia pennata*, among others.

#### Southern tropical thorn forest

This forest type is the most affected and degraded in nature and is distributed throughout the entire lower hills and foothills at <400 m above MSL, as it appears near adjoining villages. The main tree species are *Acacia planifrons*, *Acacia leucophloea*, *Albizia amara*, *Azadirachta indica*, *Streblus asper*, and *Wrightia tinctoria*, among others, and this forest type also contains heavy impenetrable thorny species such as *Carissa carandas*, *Dichrostachys cineraria*, *Pterolobium hexapetalum*, *P*. *indicum*, *Randia dumetorum*, *Toddalia asiatica*, *Euphorbia antiquorum*, and *Z*. *oenoplia*, among others.

Permission to conduct experiments was obtained from the Principle Chief Conservator of Forest, Department of Forestry, Tamil Nadu, India. Care was taken to collect soil and floristic samples without damaging the existing forest.

### Forest cover density

Forest cover density (FCD) maps of the study region were provided by the FSI as 1° × 1° tiles for academic and research purposes. Three FCD tiles of the EG in 2012 were procured on payment for this study. The spatial resolution is 24 m.

### Soil sampling and analysis

Forests were divided into 5 km × 5 km grids, excluding private land and rocky patches. In each grid, a one-soil sample location was randomly chosen and its geographical coordinate was noted. In total, 408 soil samples were collected during January–February 2012. All samples were shade-dried, pulverised with an agate mortar, and then sieved (0.2 mm). The samples were analysed following standard methods (Table 1). For microbiological studies, soil samples were collected in sterile bags and transported to the laboratory, where they were stored at 4°C until needed for further processing.

**Table 1:** Methods for soil physico-chemical and microbiological analysis.

**Estimation**	**Method**
**Physical Properties**	
**Bulk Density (BD)**	**Core method [36]**
**Chemical Properties**	
**Soil pH**	**Potentiometry 1: 25 (soil-water) [37]**
**Electrical Conductivity (EC)**	**Conductometry (1:2 soil-water suspension) [37]**
**Total Nitrogen (TN)**	**CHSN/O Elemental Analyser [38]**
**Total Potassium (TK)**	**Flame Photometry [39]**
**Total Phosphorus (TP)**	**Pemberton method [40]**
**Total Trace Elements (Fe, Mn, Zn, Cu)**	**HCl extract in Atomic Absorption Spectrophotometer (Perkin Elmer model 380) [41–43]**
**Biological properties**	
**Soil Organic Carbon (SOC)**	**CHSN/O Elemental Analyser [38]**
**Available Nitrogen (Ava N)**	**Alkaline permanganate method [44]**
**Available Phosphorus (Ava P)**	**Olsen’s extractant (0.5 M NaHCO3) at pH 8.5 [45]**
**Available Potassium (Ava K)**	**Neutral N NH4OAc method [46]**
**Microbial Biomass Carbon (MBC)**	**Fumigation extraction method [47]**
**Microbial Biomass Nitrogen (MBN)**	**Fumigation extraction method [47]**
**Total and Functional Microbes**	**Pour plate method [48]**

### Forest soil quality index

In this study, the forest soil quality was examined using 22 soil physico-chemical and biological properties. These 22 parameters were then used to calculate the forest soil quality index (FSQI). The FSQI can effectively interpret multivariate data sets, which are characteristic of soil data; a single score is able to identify an overall trend among potentially conflicting indicators [49]. The FSQI was calculated using the following steps: 1) identification of a minimum data set (MDS) of indicators, or variables that best represent soil functions associated with the selected management goal, 2) normalisation of the MDS indicators, and 3) integration of the indicator scores into an overall index of soil quality, which are each described below.

#### MDS identification

Principal component analysis (PCA) was employed in IBM SPSS version 20.1 (IBM SPSS statistics for windows, version 22.0. Armonk, NY: IBM Corp) [50] as a data reduction technique to identify the appropriate indicators without losing the vital information [51, 52]. The principle components (PCs) are linear combinations of variables that account for the maximum variance in a data set and are more accurate descriptors of the data than the variables themselves. For this study, PCs with eigenvalues ≥1.0 (>10% of the total variance explained) was taken into consideration for indexing. Highly weighed variables (eigenvectors ≥ ± 0.7) within each PC were considered significant and retained for the FSQI [24, 53].

#### Normalisation of the indicators

As the selected soil parameters were measured in heterogeneous units, they were standardised to unit-less values between 0 and 1 [32]. Depending on whether a higher value was constructive or detrimental to soil quality, indicators were arranged in ascending or descending order [32, 33]. The resultant outputs were called *Indicator Scores*. These normalised scores were calculated using the following two formulas. Eq 1 was used for indicators that tended to increase soil quality as the values increased:

$$S_{i\;normalized}=\frac{S_{i}-S_{i\;min}}{S_{i\;max}-S_{i\;min}}$$ (1)

Eq 2 was used for indicators that tended to decrease soil quality as the values increased:

$$S_{i\;normalized}=\frac{S_{i\;max}-S_{i}}{S_{i\;max}-S_{i\;min}}$$ (2)

where *Si*_*max*_ is the maximum value of the soil indicator variable, *Si* is the actual observed value, and *Si*_*min*_ is the minimum value of the soil indicator variable.

#### Calculating the PCA-FSQI

After normalisation, the MDS variable of each observed value was weighed using the PCA-derived outcome. The weighing factor was calculated via dividing the percentage of each selected MDS variable by the total percentage of variation in all PCs with eigenvectors >1. The scored indicator for each observation was calculated with the following equation:

PCAFSQI=∑Wi*Si (3)

where *Wi* is the PCA-derived weighed factor and *Si* is the normalised scores of the indicator *i*. Higher index scores indicate better soil quality.

### Spatial data analysis

Spatial autocorrelation was performed using a semi-variogram that considered all sampling points to test the spatial autocorrelation between points. The global Moran’s I test was used to estimate the overall degree of spatial autocorrelation and the analysis returns a single value, which is applicable to the entire study area [54].

### FSQI threshold and spatial mapping

To fix the FSQI threshold, the mean and 95% confidence interval (CI) of the 408 samples were calculated [55,56]:

FSQIthresholdvalue=Mean−CI (4)

The FSQI value obtained for each sample was interpolated using the inverse distance weighted method in Arc GIS 9.3. The interpolated values were grouped into two categories: degraded (FSQI < threshold value) and non-degraded (FSQI > threshold value).

### Matrix analysis and ground truth verification

Matrix analysis is a spatial process where two raster data can be verified pixel-by-pixel for its agreement. Each pixel in the resultant image will carry information from both raster. Through this method the FSI forest cover density map and the soil quality index maps were analyzed. Each pixel of resultant image showed the forest cover density and its corresponding SQI index value.

The matrix analysis between the FSQI and the FCD of FSI was performed using ERDAS IMAGINE 2011 software (Erdas Imagine, Hexgon geospatial, ink GA,USA) [57] Ground truth verification was also performed for both the FCD and FSQI to verify whether the classified areas conformed to the field data across all forest density classes.

In total there were 8 classes and for each class 25 points were randomly created and were verified on the ground using GPS. Of the total 200 quadrates, each location the vegetation data on trees and shrubs were enumerated through quadrate of 20X 20 m size covering 13 hills.

## Results and Discussion

### FCD status in the EG

**Fig 2:**
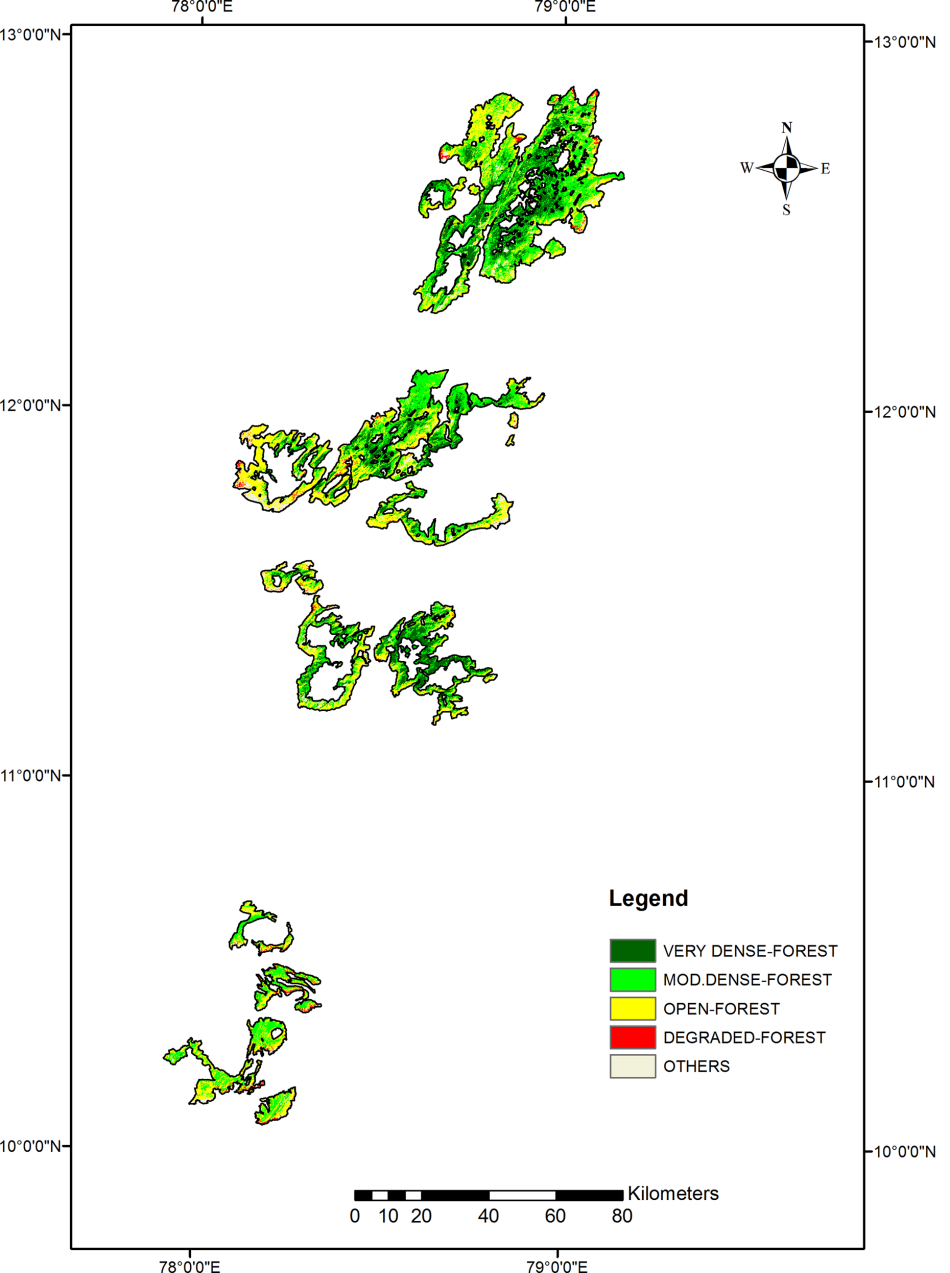
Forest cover density of the Eastern Ghats.

Table 2 details the areas of the different FCD categories. Of the site’s 422630.6 ha, approximately 45256.7 ha (10.7%) is non-forest. The effective forest area covers 377373.9 ha, with the majority categorised as moderately dense or open. Ground truth verification revealed considerable similarities between the open and the degraded forest cover categories; the floristic compositions were frequently identical, with sprawling growth of shrubs and bushes indicating heavy degradation (Table 2, Fig 2). Additionally, most of the areas occupied by scrub forests were classified as dense or moderately dense, indicating that the FSI data may be misleading and may fail to provide an accurate description of forest degradation.

**Table 2:** Forest cover density of the Eastern Ghats.

Class Names	Forest cover area (ha)	Percentage
**VERY DENSE FOREST**	66547.5	15.7
**MODERATELY DENSE FOREST**	160585.0	38.0
**OPEN FOREST**	142630.4	33.8
**DEGRADED FOREST**	7611.0	1.8
**OTHERS**	45256.7	10.7
** TOTAL**	422630.6	100.0

### FSQI

#### Soil physico-chemical and biological analysis

The physico-chemical and biological properties of the 408 soil samples are provided in Table 3. While most of the measured properties exhibited high variation, the soil pH was uniformly low. Across the 13 hills, Aiyalur displayed the lowest values for soil organic carbon (SOC), total nitrogen (TN), microbial biomass carbon (MBC), microbial biomass nitrogen (MBN), total bacteria (TB), and functional microbes. In contrast, the highest values of the measured properties were found in the Chitteri samples, which also exhibited greater variation in biological properties. Our results support previous findings that indicate that the variability in these factors is due to changes in climate, season, geographical location, and anthropogenic interference [24, 58–62].

**Table 3:** Soil physico-chemical and biological characteristics of the hills of the Eastern Ghats.

Properties	Alakar	Aiyalur	Bodamalai	Chitteri	Elagiri	Jawadi	Kalrayan	Karandai	Kolli	Pachai	Semmalai	Shevaroy	Sirumalai
**C (%)**	0.05–2.12	0.25–1.18	0.31–1.62	0.17–3.58	0.06–2.03	0.32–2.31	0.6–3.74	0.32–2.02	0.15–3.8	0.16–3.16	0.18–1.81	0.09–3.63	0.13–2.41
**N (%)**	0.1–0.45	0.07–0.53	0.04–1.02	0.09–1.33	0.11–1.17	0.1–0.9	0.11–1.43	0.04–0.85	0.02–0.85	0.01–0.65	0.11–0.43	0.05–1.30	0.07–1.02
**MBC (mg kg**^**-1**^**)**	72.9–616	142–381	80–348	40–634	72–601	72–616	111–515	110–381	83–532	83–588	132–515	24–617	130–522
**MBN (mg kg**^**-1**^**)**	67–340	26–386	57–493	39–501	43–471	45–494	23–501	45–452	24–424	56–471	94–361	11–501	56–469
**TB (cfu mL**^**-1**^**)**	1.2×10-3-7×10^7^	1.8×10^3^−3.4×10^5^	1.6×10^3^−4.5×10^6^	1.9×10^3^−4.5×10^5^	2.5×10^3^−1.2×10^5^	2.4×10^3^−4.5×10^5^	1×10^4^−4×10^6^	4.2×10^3^−8.6×10^5^	2.7×10^3^−1.2×10^5^	1.5×10^3^–3.1×10^5^	2.4×10^3^−6.5×10^4^	1.4×10^3^−5.2×10^5^	2.9×10^3^−5×10^6^
**TF (cfu mL**^**-1**^**)**	2.5×103–1.4×10^4^	2.6×10^3^−3.5×10^5^	1.1×10^3^−3.5×10^5^	1.2×10^3^−3.1×10^5^	1×10^4^−3.1×10^6^	3.2×10^3^−4.6×10^5^	2.2×10^3^−3.6×10^5^	3.1×10^3^−3.6×10^5^	3.1×10^3^−3.6×10^5^	3.2×10^3^−6.6×10^5^	3.1×10^3^−8.6×10^5^	4.2×10^3^−3.6×10^5^	4.2×10^3^−2.1×10^5^
**TA (cfu mL**^**-1**^**)**	1×10-3-3.1×10^5^	1.5×10^3^−3×10^4^	1×10^3^−4×10^5^	1×10^3^−5×10^4^	2×10^3^−1×10^4^	4×10^3^−5×10^5^	1×104–4×10^5^	2×10^3^−6×10^4^	2×10^3^−1×10^4^	1×10^3^–3×10^4^	4×10^3^−5×10^4^	4×10^3^−2×10^5^	2×10^3^−5×10^5^
**NFB (cfu mL**^**-1**^**)**	2.5×103–1.4×10^4^	2.6×10^3^−3.5×10^5^	1.1×10^3^−3.5×10^5^	1×10^3^−3×10^5^	1×10^4^−3×10^6^	3×10^3^−4×10^5^	2×10^3^−3×10^5^	3×10^3^−4×10^5^	1×10^3^−3×10^5^	2×10^3^−6.6×10^5^	1×10^3^−6×10^5^	2×10^3^−3×10^5^	2×10^3^−1×10^5^
**CM (cfu mL**^**-1**^**)**	1.2×10-3-7×10^5^	1.8×10^3^−3.4×10^4^	1.6×10^3^−4.5×10^5^	3×10^3^−4.5×10^4^	5×10^3^−1.2×10^4^	4×10^3^−4×10^5^	1×10^4^−4×10^5^	1.2×10^3^−2.3×10^5^	2×10^3^−1×10^4^	1×10^3^–3×10^5^	2×10^3^−6×10^4^	1×10^3^−2×10^4^	9×10^3^−5×10^4^
**PSB (cfu mL**^**-1**^**)**	2.5×103–1.4×10^4^	2.6×10^3^−3.5×10^5^	1.1×10^3^−3.5×10^5^	1.2×10^3^−3.1×10^4^	1×10^4^−3.1×10^5^	3.2×10^3^−4.6×10^5^	2.2×10^3^−3.6×10^4^	3.1 ×101^3^−3.6×10^4^	3.1×10^3^−3.6×10^4^	3.2×10^3^−6.6×10^4^	3.1×10^3^−8.6×10^5^	4.2×10^3^−3.6×10^4^	4.2×10^3^−2.1×10^5^
**pH**	6.2–6.7	6.1–6.7	6.1–6.9	6.1–6.7	6.5–6.71	6.3–6.8	6.1–6.7	6.1–6.5	6.0–6.4	6.2–6.7	6.2–6.7	6.3–6.9	6.0–6.4
**EC**	0.11–0.12	0.12–0.13	0.12–0.14	0.11–0.13	0.11–0.13	0.11–0.12	0.12–0.14	0.11–0.12	0.11–0.13	0.11–0.12	0.11–0.13	0.14–0.16	0.14–0.16
**TK (%)**	9–21	10–21	10–25	21–23	9–19	12–23	13–31	10–34	20–30	16–24	17–31	12–23	16–20
**TP (%)**	15–20	17–30	21–34	19–27	11–23	14–17	12–28	15–32	12–29	16–31	14–17	13–31	12–21
**Fe (mg kg**^**-1**^**)**	2.06–4.01	2.05–4.91	1.73–3.4	1.7–2.56	1.12–4.5	1.23–5.6	1.78–2.39	1.63–4.51	1.34–3.67	.1.29–3.8	1.45–2.6	1.6–4.11	1.57–4.78
**Mn (mg kg**^**-1**^**)**	4.7–9.3	5.1–9.8	3.89–9.0	1.9–10.1	2.3–8.9	3.4–9.81	2.46–8.91	3.13–8.93	3.6–8.9	4.12–9.23	3.81–8.92	3.79–10.1	4.51–7.67
**Zn (mg kg**^**-1**^**)**	1.23–2.45	1.11–2.89	1.45–2.39	1.34–2.91	1.11–3.1	1.9–2.7	1.3–2.0	1.3–2.1	1.4–2.5	1.4–3.6	1.3–2.2	1.31–2.6	1.5–2.7
**Cu (mg kg**^**-1**^**)**	1.2–2.1	1.2–2.4	1.24–2.5	1.31–4	1.34–2.1	1.21.2.1	1.67–2.3	1.11–2.2	1.31–2.0	1.1–2.0	1–2.4	1.3–3.2	1.4–2.4
**Ava N (kg ha**^**-1)**^	121–392	153–212	158–234	123–301	213–312	124–312	214–378	156–301	145–294	156–345	156–291	239–315	120–234
**Ava P (kg ha**^**-1**^**)**	15–25	17.9–59	23–34	28–79	19–67	14–65	34.5–98	13.62–78.1	11.34–70.23	19.4–72.3	23.1–78	32.4–90.1	12.7–36.7
**Ava K (kg ha**^**-1**^**)**	123–234	189–210	120–534	149–560	137–589	131–601	123–467	290–601	312–421	172–390	138–560	278–670	145–346

SOC: soil organic carbon, TN: total nitrogen, MBC: microbial biomass carbon, MBN: microbial biomass nitrogen, TB: total bacteria, TF: total fungi, TA: total actinomycetes, NFB: nitrogen fixing bacteria, CM: cellulytic microbes, PSB: phosphate solubilising bacteria, EC: electrical conductivity, TK: total potassium, TP: total phosphorus, Fe: iron, Mn: manganese, Zn: zinc, Cu: copper, Ava N: available nitrogen, Ava P: available phosphorus, Ava K: available potassium.

### Spatial data analysis

A fitted semi-variogram of the FSQI in the study area had a nugget effect of 0.55 and a total sill of 0.74. The high nugget-sill ratio of 74% reveals that strong local scale variation prevails. The spatial autocorrelation that was performed based on the global Moran’s I of the FSQI was 0.216, which was low but still statistically significant (p = 0.000014). This indicates that spatial clusters of the FSQI may be found across the study area with similar values (S1 Fig).

#### MDS

The results of our PCA on the 22 soil parameters (Table 3) revealed that the first four PCs explained approximately 80.45% of the soil variability. Further, the first two PCs accounted for approximately 57.17% of the variance, strongly indicating that the highly weighed variables in these two components can be used to assess the soil quality of this area.

Under PC1, these variables were SOC, MBC, TB, and TN; under PC2, NFB was the highest weighed value. These five properties indicate the level of soil disturbance since biological properties react relatively quickly to small changes in the soil environment and it has been broadly reported that any alteration in soil management and land use is reflected through soil properties [63–64]. Thus, this study has considered these five biological properties as the MDS for identifying the soil quality status in all 13 hills (Table 4, S2 Fig). We then used Eq 3 to calculate the weighting factors for the variables in each PC, which allowed us to obtain the FSQI.

**Table 4:** Soil parameters incorporated in the principal component analysis.

Properties	Component				
	1	2	3	4	5
**Eigen Value**	3.538	1.607	1.086	1.009	0.871
**Variance (%)**	39.31	17.86	12.067	11.217	8.910
**Cumulative (%)**	39.31	57.17	69.237	80.454	89.364
**Eigen Vectors**					
**Bulk Density**	.011	.060	.341	.213	.324
**SOC**	**.905**	.211	-.142	-.115	.211
**TN**	**.718**	-.532	.313	.175	.456
**MBC**	**.826**	.168	-.126	-.041	.567
**MBN**	.680	-.561	.263	.249	.021
**TB**	**.751**	.368	-.259	-.113	.121
**TF**	.212	.031	.191	.304	.291
**TA**	.447	.152	.596	-.437	.128
**NFB**	.310	.**775**	-.200	.527	.235
**CM**	-.239	.011	.456	.435	.146
**PSB**	.230	.111	.231	.342	.417
**pH**	.141	.212	.303	-.003	.239
**EC**	.165	.342	-.574	-.411	.454
**TK**	.344	.136	.343	.291	.157
**TP**	.034	.458	.023	.359	.478
**Fe**	.450	-.122	-.228	-.452	.234
**Mn**	-.211	.042	-.245	.127	.458
**Zn**	-.220	.124	.284	.116	.171
**Cu**	.175	.571	.466	.376	.342
**Ava N**	.178	.377	.457	.125	.561
**Ava P**	.132	.375	.399	.274	.231
**Ava K**	.231	.452	.371	.312	.341

SOC: soil organic carbon, TN: total nitrogen, MBC: microbial biomass carbon, MBN: microbial biomass nitrogen, TB: total bacteria, TF: total fungi, TA: total actinomycetes, NFB: nitrogen fixing bacteria, CM: cellulytic microbes

PSB: phosphate solubilising bacteria, EC: electrical conductivity, TK: total potassium, TP: total phosphorus, Fe: iron, Mn: manganese, Zn: zinc, Cu: copper, Ava N: available nitrogen, Ava P: available phosphorus, Ava K: available potassium.

$$\begin{array}{c} \text{PCA}-\text{FSQI}=\left[\left(0.393\;\text{SOC}+0.393\;\text{TN}+0.393\;\text{MBC}+0.393\;\text{TB}+0.178\;\text{NFB}\right)/1.75\right]\\ \text{PCA}-\text{FSQI}=0.224\;\text{SOC}+0.224\;\text{TN}+0.224\;\text{MBC}+0.224\text{TB}+0.101\;\text{NFB} \end{array}$$ (5)

**Fig 3:**
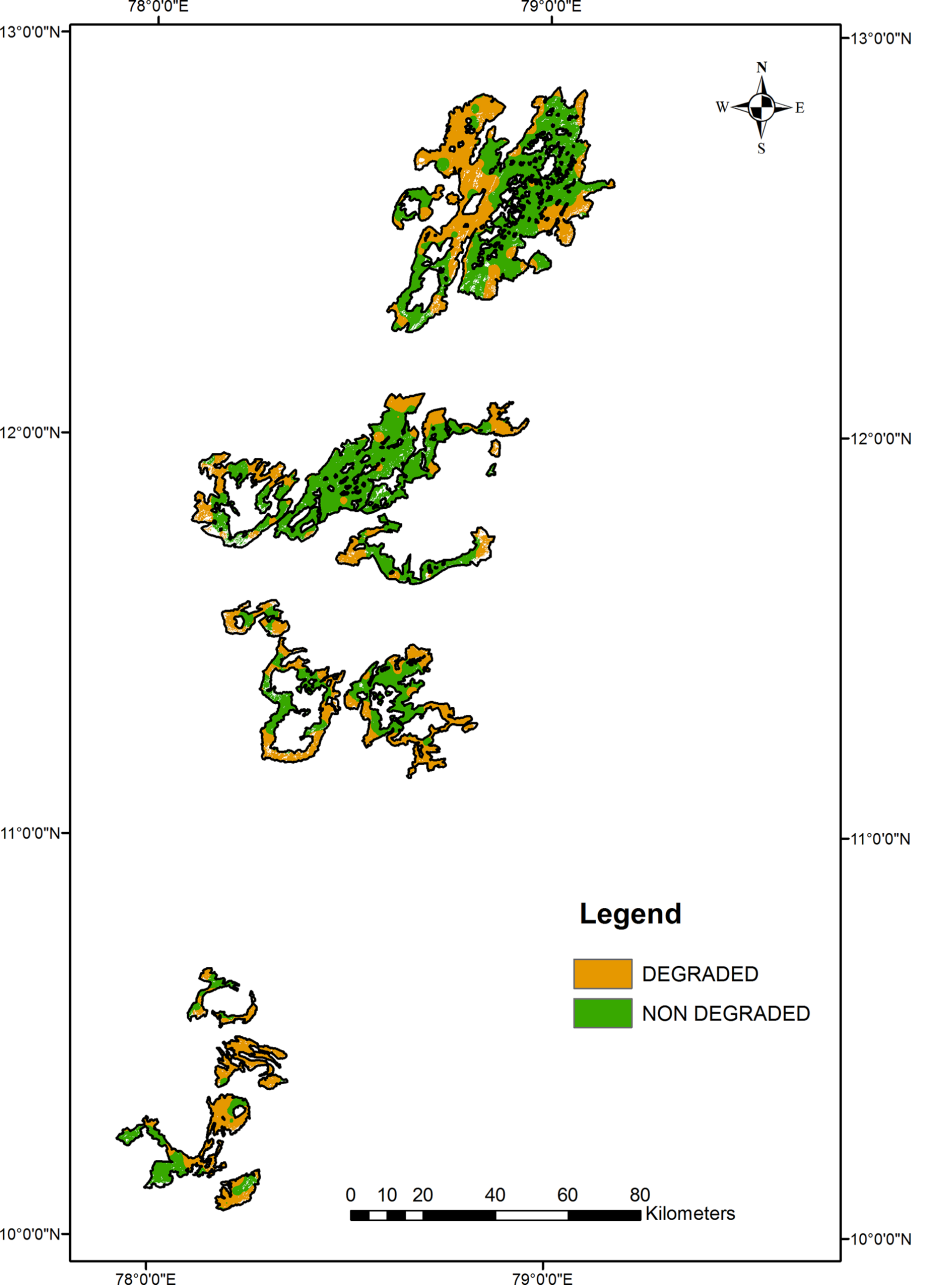
Forest soil quality index of the Eastern Ghats.

As previously stated, low FSQI values indicate degraded soil and high values indicate non-degraded soil. Soil from Chitteri Hill exhibited the highest FSQI (0.69), followed by soil from Kalrayan Hill (Table 5). Aiyalur and Bodamalai possessed soils with the lowest FSQI values: 0.32 and 0.36, respectively (Fig 3). The mean FSQI of all EG sites in Tamil Nadu was only 0.44, very close to the threshold value. Based on the FSQI, approximately 42.4% of the study area consists of degraded forests and 57.6% consists of non-degraded forests.

**Table 5:** Status of the forest soil quality index (FSQI) and areas of different forest types in the hills of the Eastern Ghats.

Hills	Total Area (ha)	Forest Cover Density (ha)		FSQI			
		Degraded	Non	Mean	Range	Degraded	Non Degraded
			Degraded			(ha)	(ha)
**Alagar**	6141.7	408	5733.7	0.38	0.13–0.67	4237.3	1904.5
**Aiyalur**	10424.1	1686	8738.1	0.32	0.21–0.50	10022.6	401.7
**Bodamalai**	7560.3	256.5	7303.8	0.36	0.13–0.77	4975.8	2584.5
**Chitteri**	49329.6	597.5	48732.1	0.69	0.17–1.24	5518.6	43810.6
**Elagiri**	6433.2	9.2	6424	0.46	0.13–1.29	2413.6	4019.6
**Jawadi**	146446.6	1788.5	144658.1	0.43	0.13–0.87	63062.9	83383.8
**Kalrayan**	41238.4	203.3	41035.1	0.56	0.20–1.38	14783.2	26455.2
**Karandai**	7877.8	360.2	7517.6	0.37	0.16–0.78	6448	1429.7
**Kolli**	23406.2	348.1	23058.1	0.42	0.09–0.81	12025	11381.2
**Pachaimalai**	34917.2	177.8	34739.4	0.41	0.10–1.05	17356.2	17560.9
**Semmalai**	6246.5	441.2	5805.3	0.39	0.16–0.58	3354.2	2892.3
**Shevaroy**	23957.2	919.2	23038	0.46	0.01–1.42	11330.6	12626.8
**Sirumalai**	13395.1	415.5	12979.6	0.46	0.17–1.20	4590.5	8804.6
**Total**	**377373.9**	**7611**	**369762.9**	** **	** **	**160118.5**	**217255.4**

However, ground truth verification revealed a ±13% deviation from the FSQI-classified status, due to variation in vegetation within a particular forest density class. For instance, the presence of riparian flora along streams in the open and degraded forests meant they were apparently classified as dense forests.

Based on the FSQI, the highest soil degradation (>96.1%) was found in Aiyalur forests, followed by Bodamalai and Karandamalai (81.9%) and Alagar (69%) forests (Table 5). Furthermore, the lowest soil degradation occurred in Chitteri, with 11.2% of the area classified under the degraded category. In addition, the soil quality of Sirumalai, Kalrayan, and Elagiri was found to be less affected by forest degradation, with 34.3%, 35.8%, and 37.5% of their total areas classified as degraded, respectively (Table 5).

**Fig 4:**
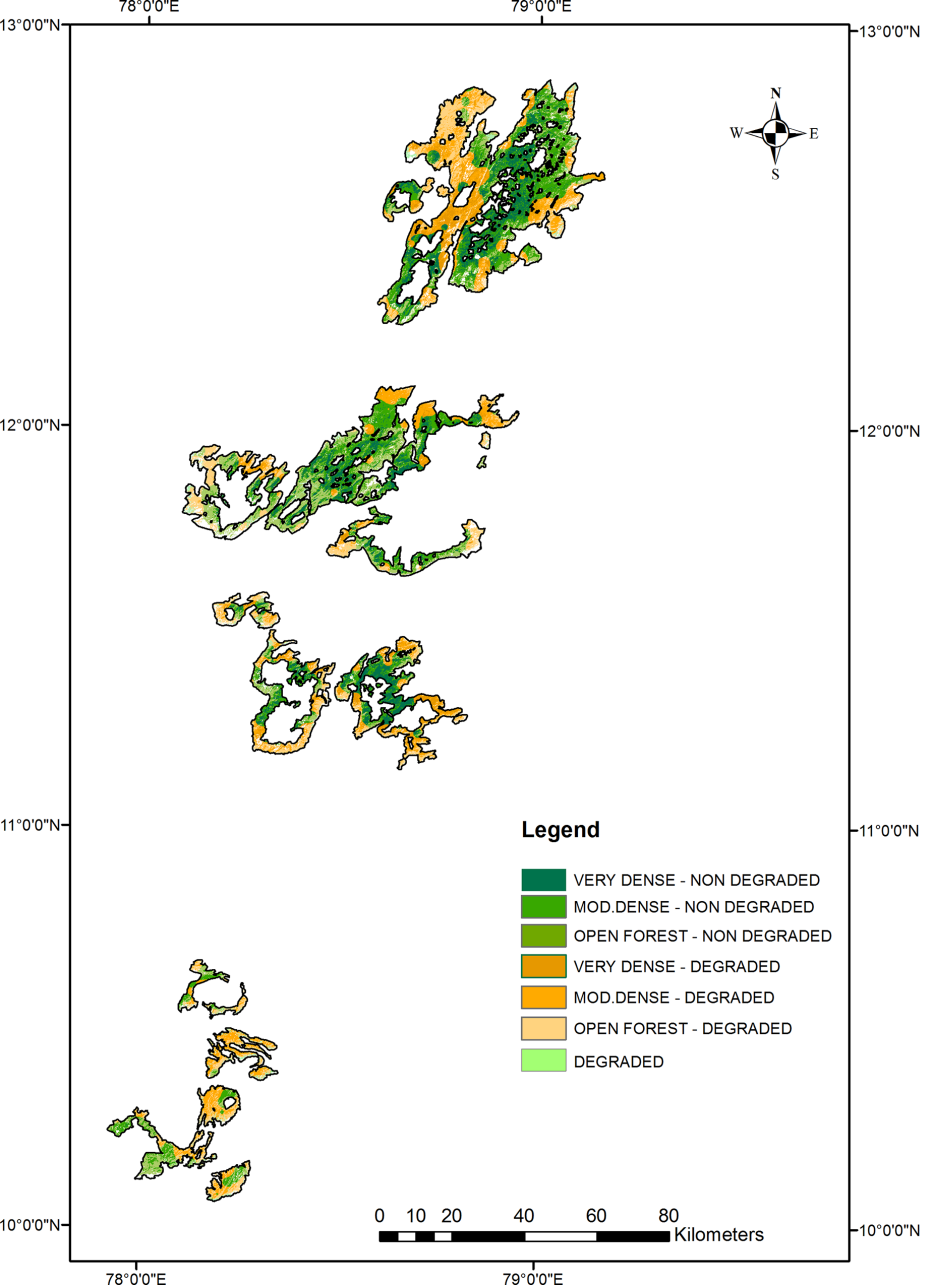
Matrix analysis between the forest cover density and forest soil quality index.

The matrix analysis of the FCD and FSQI (Table 6) revealed more details about the degradation status. Forests in all four FCD categories have both degraded and non-degraded soil. In the degraded forest category, 74.2% possesses degraded soil, and in the open forest cover category, approximately 52% possesses degraded soil. In the moderately dense and very dense categories, degraded soil occurs in 38.9% and 26.7% of the forest areas, respectively. Across all forests, only 154474.2 ha (40.9%) were classified as non-degraded by both the FCD and FSQI (Fig 4). Based on previous reports of the EG, soil quality degenerated wherever severe anthropogenic exploitation slowly modified primary forest vegetation to secondary vegetation [8, 10]. Such forests tend to consist of patchily distributed, low scrub that degenerates into spare bushes with occasional, stunted tree growth predominantly occupied by *L*. *camara* bushes that suppress the natural regeneration of other species [3, 13, 65–68].

**Table 6:** Matrix analysis between the forest cover density and forest soil quality index.

Class Name	FSI Forest	FSI and FSQI Matrix			
	cover (ha)	Degraded (ha)	Non-Degraded (ha)	Degraded (%)	Non Degraded (%)
**VERY DENSE FOREST**	66547.5	17741.4	48806.1	26.7	73.3
**MODERATELY DENSE FOREST**	160585.0	62515.5	98069.5	38.9	61.1
**OPEN FOREST**	142630.4	74217.3	68413.1	52.0	48.0
**DEGRADED**	7611.0	5644.3	1966.7	74.2	25.8
**TOTAL**	**377373.9**	**160118.5**	**217255.4**	**42.4**	**57.6**

FSI: Forest Survey of India.

Although there is some correlation between the forest density and the percentage of degradation estimated by the FSI, the present study clearly addresses the inconsistencies between the density classes of the FSI and FSQI (Table 6). Forests classified by the FSI as very dense forests, moderately dense forests, and open forests contain soils below the FSQI threshold, while high FSQI values occurred in forests classified as degraded by the FSI. Overall, an imprecise relationship exists between the FSI density classes and the FSQI. We conclude that the NDVI-based forest cover assessment cannot comprehensively describe the real degradation status of forests.

The species composition of various slope categories in the EG (Table 7) reveals two distinct patterns that correspond to the FSQI rather than the FCD. Wherever the FSQI value is below the threshold, thorny scrubs instead of trees generally dominate the floristic composition. Moreover, when the FSQI value is higher than the threshold, dense tree growth dominates, with a very limited occurrence of herbs and shrubs (Table 7). A large contributor to these patterns is the invasive species *L*. *camara* [65–68], which was extremely prominent in areas with low FSQI values and appeared to prevent further proliferation of native species. Other factors that aggravate soil degradation and hinder plant growth include anthropogenic activity, environmental factors such as erratic rainfall and extreme heat during summer, as well as geological features like steep slopes [10].

**Table 7:** Matrix-based species composition in degraded and non-degraded forests located in the lower, middle, and upper slopes, as verified through ground truth surveys.

Forest types	Species Composition	
	Degraded (Low FSQI)	Non-degraded (High FSQI)
**Thorn forest**	**Tree species:** *Moringa oleifera*,	**Tree species:** *Azadirachta*
**(Lower elevation**	*Gyrocarpus americanus*, *Wrightia*	*indica*, *Acacia sundra*,
**<400 m)**	*tinctoria*, *Acacia ferruginea*, *acacia*	*Hardwickia binata*, *Albizia*
	*leucophloea*, *Acacia sundra*,	*amara*, *Chloroxylon swietenia*,
	*Acacia planifrons*, *Albizia*	*Acacia leucophloea*, *Zizyphus*
	*Odoratissima*	*xylopyrus*, *Moringa oleifera*,
	**Shrubs:** *Lantana camara*, *Strychnos*	*Wrightia tinctoria*, *Feronia*
	*potatorum*, *Pterolobium indicum*,	*elephantum*, *Streblus asper*
	*Acacia concinna*, *Acacia latronum*,	**Shrubs:** *Dichrostachys Randia*
	*Acacia intsia*, *Dichrostachys cineraria*,	*cineraria*, *Dodonaea viscosa*,
	*Dodonaea viscosa*, *Randia dumetorum*,	*dumetorum*, *Clausena dentata*,
	*Clausena dentata*, *Commiphora*	*Commiphora caudata*, *Carissa*
	*Caudate*	*carandas*, *Acacia intsia*, *Acacia*
		*Latronum*
**Deciduous forest**	**Tree species:** *Albizia odoratissima*,	**Tree species:** *Shorea talura*,
**Middle elevation**	*Canthium didymum*, *Anogeissus*	*Anogeissus latifolia*, *Terminalia*
**(400–1000 m)**	*latifolia*, *Feronia elephantum*, *Ziziphus*	*tomentosa*, *Terminalia*
	*xylopyrus*, *Erythroxylon monogynum*	*paniculata*, *Albizia lebbeck*,
		*Albizia odoratissima*, *Terminalia*
	**Shrubs:** *Lantana camara*, *Strychnos*	*arjuna*, *Hardwickia binata*,
	*nuxvomica*, *Citronella grass*,	*Diospyros montana*,
	*Pterolobium indicum*, *Acacia intsia*,	*Stereospermum suaveolens*,
	*Pavetta indica*, *Acacia pennata*,	*Pterocarpus marsupium*, *Adina*
	*Pterolobium indicum*, *Acacia intsia*,	*cordifolia*, *Bridelia retusa*,
	*Ziziphus oenoplia*	*Dalbergia latifolia*, *Ficus*
		*microcarpa*, *Grewia tiliifolia*,
		*Premna tomentosa*, *Santalum*
		*album*, *Sapindus emarginatus*,
		*Schleichera oleosa*
		**Shrubs:** *Pterolobium indicum*,
		*Acacia intsia*, *Ziziphus oenoplia*,
		*Semecarpus anacardium*,
		*Ziziphus mauritiana*, *Lantana*
		*Camera*
**Evergreen and**	**Tree species:** *Memecylon edule*,	**Tree species:** *Manilkara hexandra*,
**semi-evergreen**	*Memecylon umbellatum*, *Persea*	*Memecylon edule*, *Alseodaphne*
**(Upper elevation >**	*macrantha*, *Melia dubia*, *Syzygium*	*semecarpifolia*, *Alstonia scholaris*,
**1000 m)**	*montana*, *Trema orientalis*	*Bischofia javanica*, *Celtis tetrandra*,
		*Chukrasia tabularis*, *Cinnamomum*
	***Shrubs*:** *Lantana camara*, *Embelia indica*,	*macrocarpum*, *Elaeocarpus*
	*basaal*, *Atlantia monophylla*, *Clausena*	*lanceifolius*, *Litsea oleoides*, *Mallotus*
	*dentata*, *Glycosmis mauritiana*, *Mesa*	*philippensis*, *Mangifera indica*,
	*Psychotria subintegra*, *Phyllanthus*	*Meliosma simplicifolia*, *Memecylon*
	*wightianus*, *Tarenna asiatica*,	*edule*, *Memecylon umbellatum*,
	*Adenostemma lavenia*, *Balanophora*	*Myristica dactyloides*, *Neolitsea*
	*fungosa*, *Dorstenia indica*	*scrobiculata*, *Nothopegia*
		*colebrookeana*
		**Shrubs:** *Gymnema kollimalayanum*,
		*Embelia basaal*, *Atlantia monophylla*,
		*Clausena dentata*, *Glycosmis*
		*Mauritiana*

FSQI: forest soil quality index.

In summary, our study lends further support to previous findings that demonstrate the impreciseness of NDVI-based forest cover assessments, which fail to differentiate between natural forests and plantations [69]. Our results strongly suggest that satellite-based assessment of canopy cover gives an incomplete idea of forest status. However, we also found that the FSQI can be an effective tool for categorising forest degradation with greater accuracy.

## Conclusions

This study pioneers the use of the FSQI for estimating forest degradation in India, which currently depends on satellite-based canopy cover data to assess forest status. Using the FSQI, degradation was found in all FSI forest cover density classifications. Combining the FSQI with the FSI density classes resulted in a more detailed picture of forest degradation. Hence, the FSQI is an ideal tool for studying the degradation status distributed across different forest types and density classes. The present study reveals that the FSQI categorised approximately 42.4% of the total forests examined as degraded, whereas the FCD only categorised approximately 1.8% of the forests as degraded. Therefore, relying on satellite data alone may no longer provide an accurate representation of forest status, given the increase in environments that may appear as undisturbed forest without a more sensitive investigation. We suggest that the FSQI can be used as supplemental data along with the FSI forest cover density to efficiently categorise forest degradation and to address this continuing environmental issue, especially in developing countries. Management of degraded forests involves meticulous reforestation work to restore the forests, which could be achieved only by prioritising the area based on the FSQI.

## Supporting Information

S1 Fig Semi- variogram model of Eastern Ghats FSQI distribution using ordinary kriging based on 408 samples collected from the Eastern Ghats. (TIF)

S2 Fig Biplots for PCA Analysis. (TIF)
