# Supplementary figures and images for: Critical Analysis of Forest Degradation in the Southern Eastern Ghats of India: Comparison of Satellite Imagery and Soil Quality Index
Source: PLoS One. 2016 Jan 26;11(1):e0147541. doi: 10.1371/journal.pone.0147541 (PMC4727793; doi:10.1371/journal.pone.0147541)

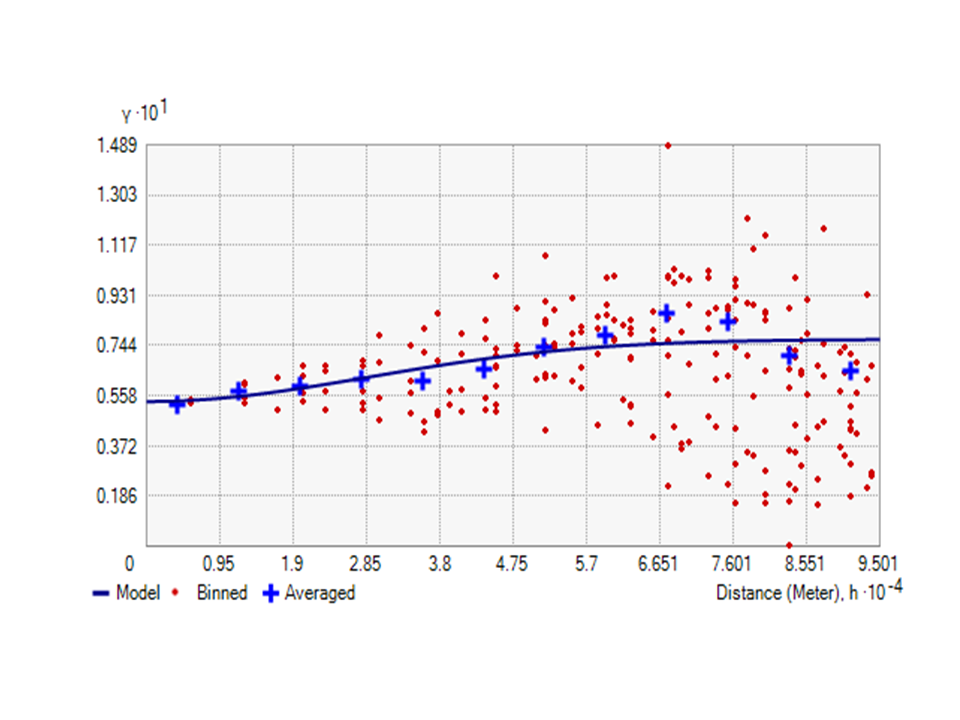

Supplement: S1 Fig — (TIF) [file pone.0147541.s001.tif]

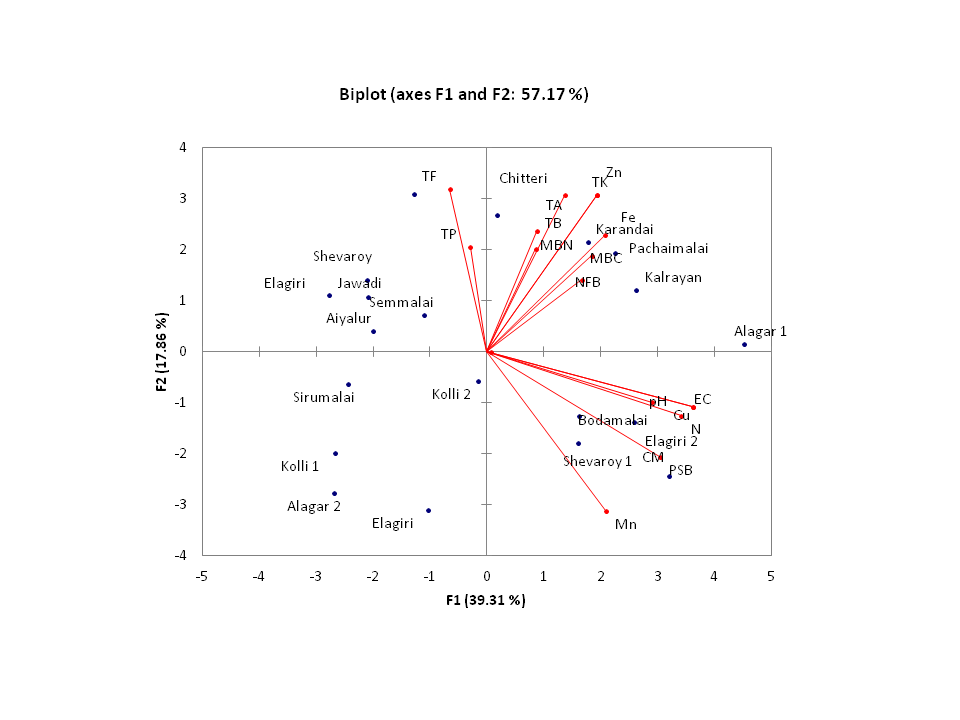

Supplement: S2 Fig — (TIF) [file pone.0147541.s002.tif]
